# How is depression in the elderly patient diagnosed?

**DOI:** 10.1192/j.eurpsy.2023.1995

**Published:** 2023-07-19

**Authors:** R. Fernández Fernández, Á. Izquierdo de la Puente, P. del Sol Calderón, O. Méndez González

**Affiliations:** 1 Psychiatry, Hospital Universitario Infanta Cristina, Parla; 2 Psychiatry, Hospital Universitario Puerta de Hierro, Majadahonda, Spain

## Abstract

**Introduction:**

The diagnosis of depression in the elderly patient presents peculiarities that should be taken into account. Studies point out the importance of an adequate screening of suspected cases of depression in older adults by physical therapists and other non-mental health professionals (Ramos Vieira et al., 2014). In this study, we intend to find out which are the most used diagnostic methods in Mental Health research on geriatric patient.

**Objectives:**

To analyze the diagnostic methods most used in research on the geriatric patient, specifically in articles that analyze the patient with cognitive impairment.

**Methods:**

A bibliographic search of all articles analyzing depression in patients with cognitive impairment between 2000 and 2020 was carried out. The diagnostic method of depression in each of them has been collected.

**Results:**

A total of 38 studies were analyzed. The most common diagnostic method continues to be the use of diagnostic criteria (ICD or DSM), which is used in 34.2% of the studies, while the Center for Epidemiologic Studies Depression Scale (CES-D) is the most commonly used test, appearing in 23.7% of the studies. The remaining tests (CIDI, GDS, HAM17, PHQ, SCID, SCL-90, SGDS) do not reach 10% each.

**Table:** 

	**Counts**	**% of Total**	**Cumulative %**
GDS	2	5.3 %	5.3 %
CES-D	9	23.7 %	28.9 %
CIDI	2	5.3 %	34.2 %
Diagnostic criteria	13	34.2 %	68.4 %
EURO-D	1	2.6 %	71.1 %
PHQ	2	5.3 %	76.3 %
GMS-AGECTA	2	5.3 %	81.6 %
HAM-17	1	2.6 %	84.2 %
Others	6	15.8 %	100 %

**Conclusions:**

The diagnosis of depression continues to be made primarily using diagnostic criteria. It is striking that the most commonly used test is the CES-D, given that the Geriatric Depression Scale (GDS) is usually the most popular scale for screening for late-life depression (Gana et al., 2017), which may be due to the fact that the studies analyzed have a more research than clinical purpose.

**References:**

Gana, K., Bailly, N., Broc, G., Cazauvieilh, C., & Boudouda, N. E. (2017). The Geriatric Depression Scale: does it measure depressive mood, depressive affect, or both?. International journal of geriatric psychiatry, 32(10), 1150–1157.

Vieira, E. R., Brown, E., & Raue, P. (2014). Depression in older adults: screening and referral. Journal of geriatric physical therapy (2001), 37(1), 24–30.

**Disclosure of Interest:**

None Declared

